# Investigation on the Electrochemical Performances of Mn_2_O_3_ as a Potential Anode for Na-Ion Batteries

**DOI:** 10.1038/s41598-020-66148-w

**Published:** 2020-06-08

**Authors:** Nor Fazila Mahamad Yusoff, Nurul Hayati Idris, Muhamad Faiz Md. Din, Siti Rohana Majid, Noor Aniza Harun, Md. Mokhlesur Rahman

**Affiliations:** 1 Energy Storage Research Group, Faculty of Ocean Engineering Technology and Informatics, Universiti Malaysia Terengganu, 21030 Kuala Nerus, Terengganu Malaysia; 2 Department of Electrical and Electronic Engineering, Faculty of Engineering, National Defence University of Malaysia, Kem Sungai Besi, 57000 Kuala Lumpur Malaysia; 3 Center for Ionics University of Malaya, Department of Physics, Faculty of Science, University of Malaya, 50603 Kuala Lumpur, Malaysia; 4 Advance Nano Materials (ANOMA) Research Group, Faculty of Science and Marine Environment, Universiti Malaysia Terengganu, 21030 Kuala Nerus, Terengganu Malaysia; 5 Institute for Frontier Materials, Deakin University, Waurn Ponds, Victoria 3216 Australia

**Keywords:** Energy storage, Batteries, Materials for energy and catalysis, Batteries

## Abstract

Currently, the development of the sodium-ion (Na-ion) batteries as an alternative to lithium-ion batteries has been accelerated to meet the energy demands of large-scale power applications. The difficulty of obtaining suitable electrode materials capable of storing large amount of Na-ion arises from the large radius of Na-ion that restricts its reversible capacity. Herein, Mn_2_O_3_ powders are synthesised through the thermal conversion of MnCO_3_ and reported for the first time as an anode for Na-ion batteries. The phase, morphology and charge/discharge characteristics of Mn_2_O_3_ obtained are evaluated systematically. The cubic-like Mn_2_O_3_ with particle sizes approximately 1.0–1.5 µm coupled with the formation of Mn_2_O_3_ sub-units on its surface create a positive effect on the insertion/deinsertion of Na-ion. Mn_2_O_3_ delivers a first discharge capacity of 544 mAh g^−1^ and retains its capacity by 85% after 200 cycles at 100 mA g^−1^, demonstrating the excellent cyclability of the Mn_2_O_3_ electrode. Therefore, this study provides a significant contribution towards exploring the potential of Mn_2_O_3_ as a promising anode in the development of Na-ion batteries.

## Introduction

Sodium-ion (Na-ion) batteries have been introduced as a possible alternative to lithium-based ion batteries due to several reasons, including abundant supply, low cost and less toxicity^1,2^. However, sodium cannot be simply swapped with lithium as sodium has a larger ion size (1.02 Å for Na^+^ as compared to 0.76 Å for Li^+^) and slightly different chemistry, resulting in sluggish reaction kinetics that usually causes low capacity, poor rate capability and poor cyclability^1,3–5^. Therefore, challenges still exist to find suitable anode materials for the development of Na-ion batteries and scientists are searching for the best material among a vast number of materials using the trial-and-error approach.

Among the various types of materials, metal oxides have been explored extensively for lithium-ion (Li-ion) batteries. Similar to Li-ion batteries, metal oxides can potentially be used as large capacity anodes for Na-ion batteries because of their high theoretical capacities resulting from conversion reaction in most cases. For example, one-step conversion reaction of metal oxide with Na-ions can deliver high theoretical specific capacities of >600 mAh g^−1^ according to the reaction described in Eq. (1) ^6,7^.

1 $${{\rm{M}}{\rm{O}}}_{x}+2x{\rm{N}}{\rm{a}}+2x{{\rm{e}}}^{-}\leftrightarrow x{{\rm{N}}{\rm{a}}}_{2}{\rm{O}}+{\rm{M}}({\rm{M}}={\rm{C}}{\rm{o}},\,{\rm{F}}{\rm{e}},\,{\rm{M}}{\rm{n}},\,{\rm{C}}{\rm{u}},\,{\rm{e}}{\rm{t}}{\rm{c}}.)$$

Among different conversion-type transition metal oxides for anodes, manganese oxides exhibit advantages of high capacity, natural richness, low cost and environmental benignity. Even though manganese oxides including mono, Mn_3_O_4_, Mn_2_O_3_, MnO_2_ and their carbon-based composites with different nanostructures have been found technologically important in Li-ion batteries^8^, the use of manganese oxides in Na-ion batteries is rarely reported. In 2014, Jiang *et al*.^9^ synthesised Mn_3_O_4_ and investigated its reactivity as anode towards sodium for the first time. Subsequently, Weng *et al*.^10^ prepared MnO_2_ using a SiO_2_-templated hydrothermal approach, which was used as conversion-type anode for Na-ion batteries. However, rapid irreversible fading of capacities following the cycling process is a common problem with MnO_2_ and Mn_3_O_4_ phases due to volume expansion and aggregation as well as low electronic conductivity. To tackle these problems with transition metal oxide anodes, nanostructure engineering is widely adopted, where structural parameters including particle size, crystal size and morphology act as critical factors in achieving maximum electrochemical performance. By adopting nanostructure engineering, much improvement of sodium storage properties was realised with MnO_2_ anode by the development of new structures such as MnO_2_ nanorods and nanoflowers^11,12^.

In this study, Mn_2_O_3_ is synthesised by combining a hydrothermal and a thermal decomposition method and used as Na-ion battery anode for the first time. We developed a cubic structure of Mn_2_O_3_ by simple thermal decomposition of manganese carbonate (MnCO_3_) precursor through controlled calcination temperatures. The crucial feature of this structure is that cubic particles of Mn_2_O_3_ are approximately 1–2 µm in size and are composed of numerous nanoparticles (sub-units) of 40–50 nm grown on the surface, leading to more accessible sites for electrolyte penetration into the bulk of the electrode, which facilitates ion transportation thereby promoting the insertion/deinsertion of Na-ions^13^. The obtained Mn_2_O_3_ anode prepared at 600 °C demonstrates an impressive capacity of 130 mAh g^−1^ at 100 mA g^−1^ after 200 cycles with a remarkable rate capability of 120 mAh g^−1^ at a very high current of 1000 mA g^−1^ even without any cation doping or carbon coating.

## Results and Discussion

**Figure 1:**
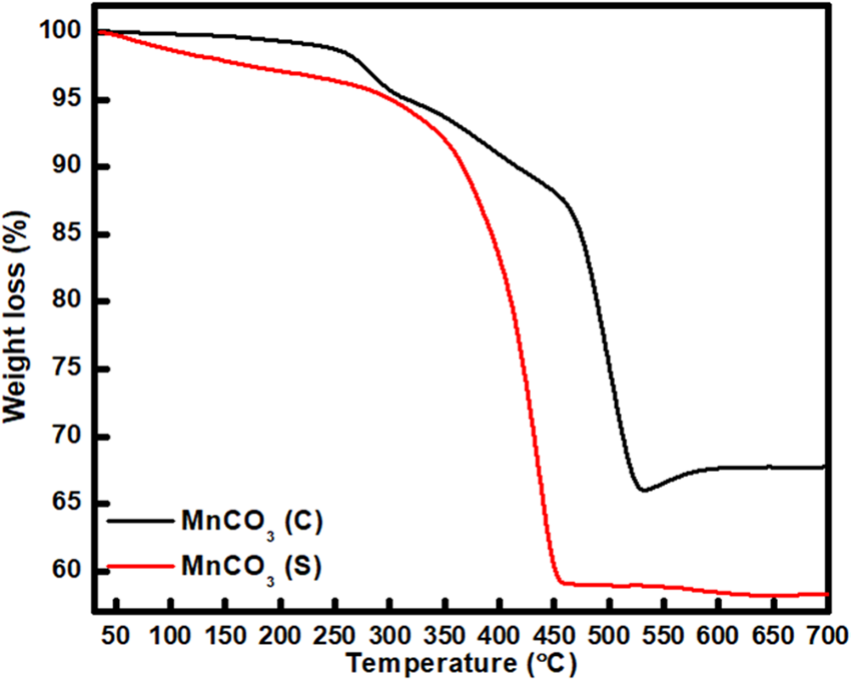
TGA curves of MnCO_3_ (C) and MnCO_3_ (S) at a heating rate of 10 °C min^−1^ in air.

To verify the nature of decomposition and the formation temperature of Mn_2_O_3_, TGA analysis of both MnCO_3_ precursors (synthesized MnCO_3_ (denoted as MnCO_3_ (S) and commercially available MnCO_3_ (MnCO_3_ (C)) was performed. It is clearly observed that the nature of decomposition of MnCO_3_ (S) and MnCO_3_ (C) precursors within the same temperature range is different, as shown in Fig. 1. One-step decomposition is realised with MnCO_3_ (S), whereas MnCO_3_ (C) precursor shows the two-step decomposition. Generally, the initial undulation appeared with initial weight loss of ~3 wt.% caused by the loss of hydrated water from MnCO_3_. The weight loss appeared between 250 and 350 °C for MnCO_3_ (C), presumably due to the formation of MnO_2_ which is further confirmed by XRD analysis (Fig. S1). The commercial MnCO_3_ (C) was heated up to temperature 300 °C in air for 2 h and the obtained product was analysed by XRD. The XRD pattern shows the presence of MnO_2_ and MnCO_3_ (C) in the product, suggesting partial decomposition of MnCO_3_ (C) and formation of MnO_2_^14^. It is obvious that the weight loss between 400 and 600 °C is attributed to the formation of Mn_2_O_3_ and the release of O_2_^14–16^. Since only one-step decomposition slope is observed with MnCO_3_ (S), the possible reaction during the decomposition of MnCO_3_ (S) can be summarised as Eq. (2) ^17,18^:

2 $$2{{\rm{MnCO}}}_{3}+{{\rm{O}}}_{2}\to {{\rm{Mn}}}_{2}{{\rm{O}}}_{3}+2{{\rm{CO}}}_{2}$$

Reducing the decomposition step of MnCO_3_ (S) indicates that the conversion process is favourable since the kinetic process is shortened to obtain clear facet of Mn_2_O_3_.

**Figure 2:**
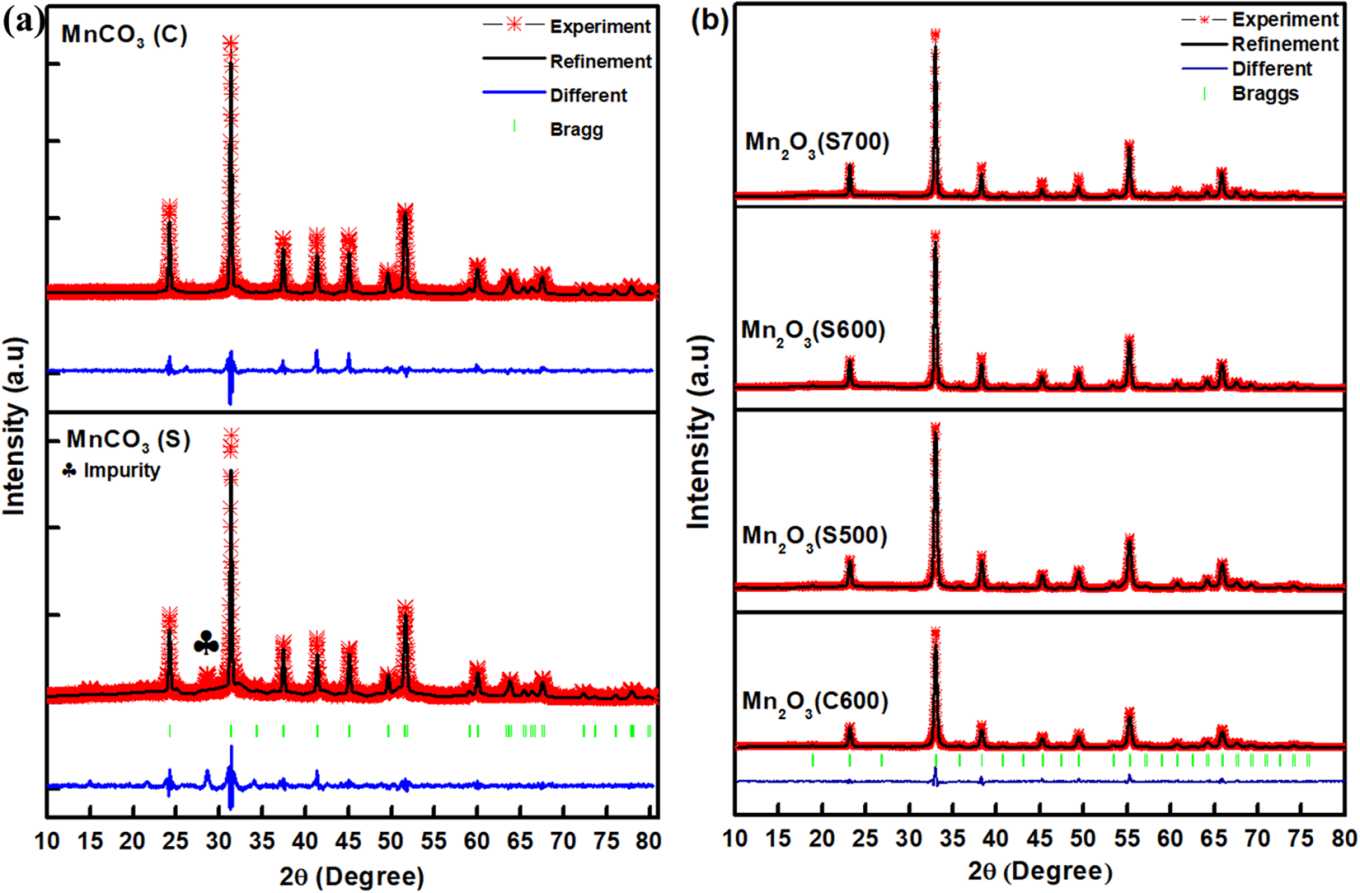
Rietveld refinement fits of the XRD data: (**a**) Commercial and synthesized MnCO_3_; and (**b**) Mn_2_O_3_ (C600), Mn_2_O_3_ (S500), Mn_2_O_3_ (S600) and Mn_2_O_3_ (S700) powders.

Figure 2 shows the XRD patterns along with the Rietveld refinement profiles for MnCO_3_ and Mn_2_O_3_. Figure 2(a) shows the refinement patterns for MnCO_3_ (C) and MnCO_3_ (S) precursors. All the diffraction peaks could be indexed to the rhodochrosite phase of MnCO_3_ (JCPDS card No: 044-1472) with *R-3c* space group (rhombohedral unit cell). The refined lattice parameters for MnCO_3_ (C) were *a* = 4.8045 (2) Å, *c* = 15.6892 (8) Å, and the unit cell volume was 313.636 Å^3^ (Table S1). For MnCO_3_ (S), the lattice parameters were smaller than MnCO_3_ (C) with *a* = 4.8019 (3) Å, *c* = 15.6798 (1) Å and a unit cell volume of 313.103 Å^3^ that are consistent with the previous report^19^. However, a small diffraction peak appeared at 2θ value of 28.5° for MnCO_3_ (S), which is attributed to impurity that could have formed due to incomplete utilisation of manganese precursor during the hydrothermal process^20^. Furthermore, the average crystallite sizes (*L*) of MnCO_3_ were calculated according to the Scherrer’s equation as shown in Eq. (3):

3 $$L=\frac{k}{\beta \,\cos \,\theta }$$

where, *k* is the constant (0.9394), *λ* is the X-ray wavelength of Cu-Kα (1.5148 Å), *β* is the FWHM of the XRD peak in radian and *θ* is the angle of diffraction. The calculated average crystallite sizes of the MnCO_3_ (C) and MnCO_3_ (S) samples were ~36 nm and ~35 nm, respectively, which is in good agreement with reported result^21^. Figure 2(b) shows quantitative analysis of the Rietveld refinement fit profiles along with the observed XRD patterns of Mn_2_O_3_ (C600), Mn_2_O_3_ (S500), Mn_2_O_3_ (S600) and Mn_2_O_3_ (S700) samples. All the diffraction peaks matched well and could be indexed to a cubic Mn_2_O_3_ with the space group of *la-3* (JCPDS card no.041-1442). No other diffraction peaks of any impurities have been detected. As the calcination temperature increases, the intensity of the diffraction peaks of Mn_2_O_3_ increased, indicating improved crystallinity^22^. No changes were observed in the crystallite size of Mn_2_O_3_ (~23 nm) at the calcination temperature of 500 and 600 °C, whereas it was ~31 nm at 700 °C based on the (222) peak. Nevertheless, crystallite size decreases during transformation of MnCO_3_ to Mn_2_O_3_. The details of lattice parameter, goodness of fit and other related fitting parameters of Mn_2_O_3_ obtained from crystal structure refinement are consistent with other reports^23,24^. Clearly, the lattice parameter of Mn_2_O_3_ increases as the calcination temperature increases due to the expansion during crystal growth.

**Figure 3:**
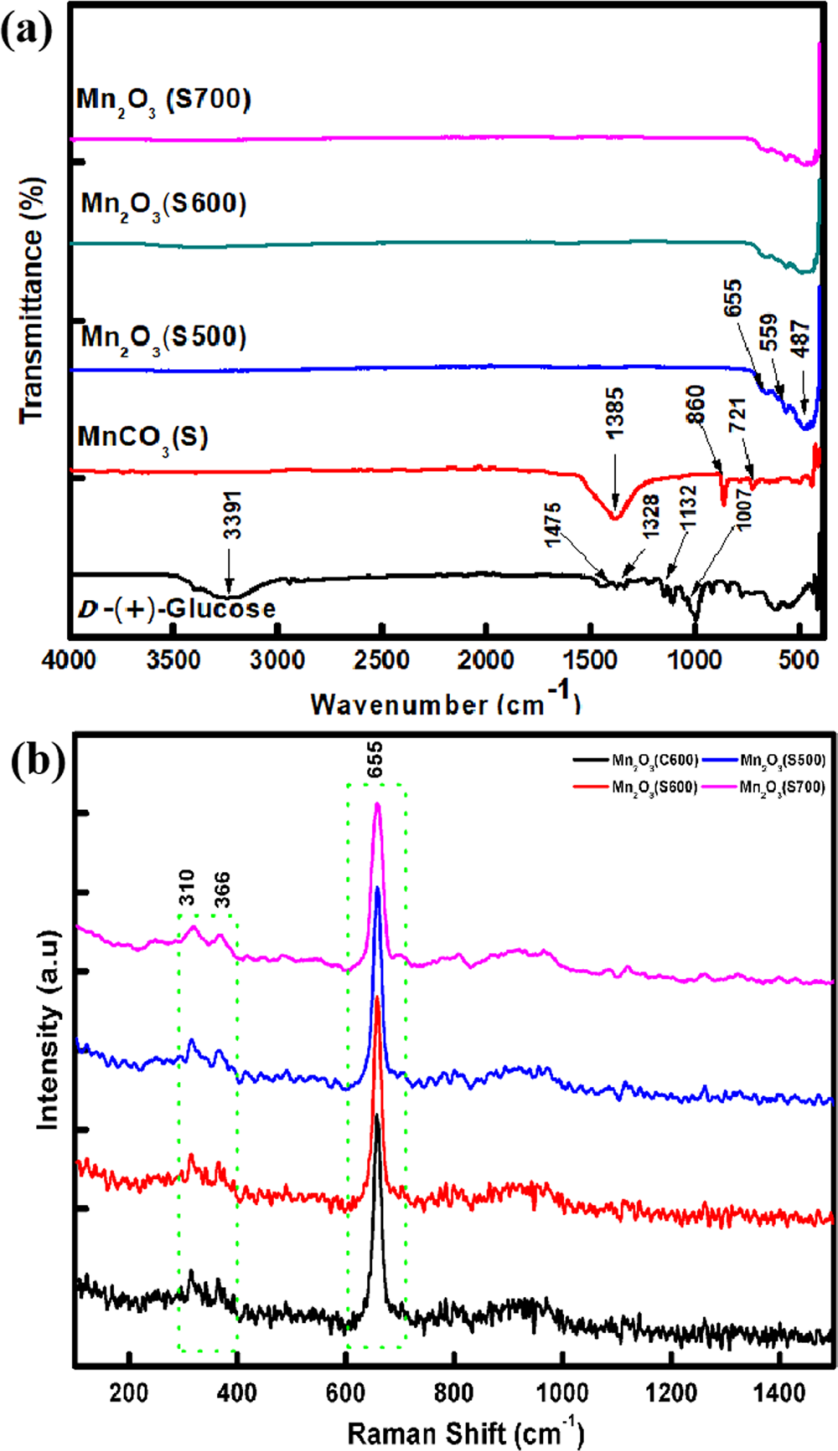
(**a**) FTIR spectra of D-glucose, MnCO_3_ (S)_,_ Mn_2_O_3_ (S500), Mn_2_O_3_ (S600) and Mn_2_O_3_ (S700); and (**b**) Raman spectra of Mn_2_O_3_ (C600), Mn_2_O_3_ (S500), Mn_2_O_3_ (S600) and Mn_2_O_3_ (S700).

Figure 3(a) shows FTIR spectra of D-glucose, MnCO_3_ and Mn_2_O_3_ samples. The FTIR spectrum of D-glucose shows the existence of a strong and broad absorption peak at 3391 cm^−1^ indicating the presence of *v*(OH) group stretching vibration. A small peak at 2920 cm^−1^ was attributed to the absorption peak of *v*(CH_2_) group, and the bands at 1475 cm^−1^ and 1328 cm^−1^ were assigned to the bending vibration of *v*(CH). The *v*(C–O) and *v*(C–C) stretching bands were observed at 1132 and 1007 cm^−1^, respectively^25,26^. During hydrothermal process, the D-glucose peaks (O–H bond at 3391 cm^−1^ and C–H bond at 1475 cm^−1^) were completely vanished due to the formation of MnCO_3_ (S) with the presence of C–O bending vibration of carbonate peaks at 1384, 860 and 721 cm^−1^ ^27^. When MnCO_3_ was heated at high temperature, the carbonate peaks diminished. The presence of three absorption peaks located at 487, 559 and 655 cm^−1^ for Mn_2_O_3_ may be attributed to Mn–O and Mn–O–Mn, confirming the formation of Mn_2_O_3_^25,27,28^, which is consistent with the Raman spectroscopy results as shown in Fig. 3(b). Raman active bands located at 170–1000 cm^−1^ may be due to the Mn–O vibration modes of manganese oxides^29,30^. The Raman bands at 310, 366 and 655 cm^−1^ are corresponding to the bending modes of Mn_2_O_3_, the asymmetric stretch of Mn–O–Mn and an asymmetric stretch of Mn_2_O_3_ corresponding to Mn (III)–O mode vibration group, respectively^25^.

**Figure 4:**
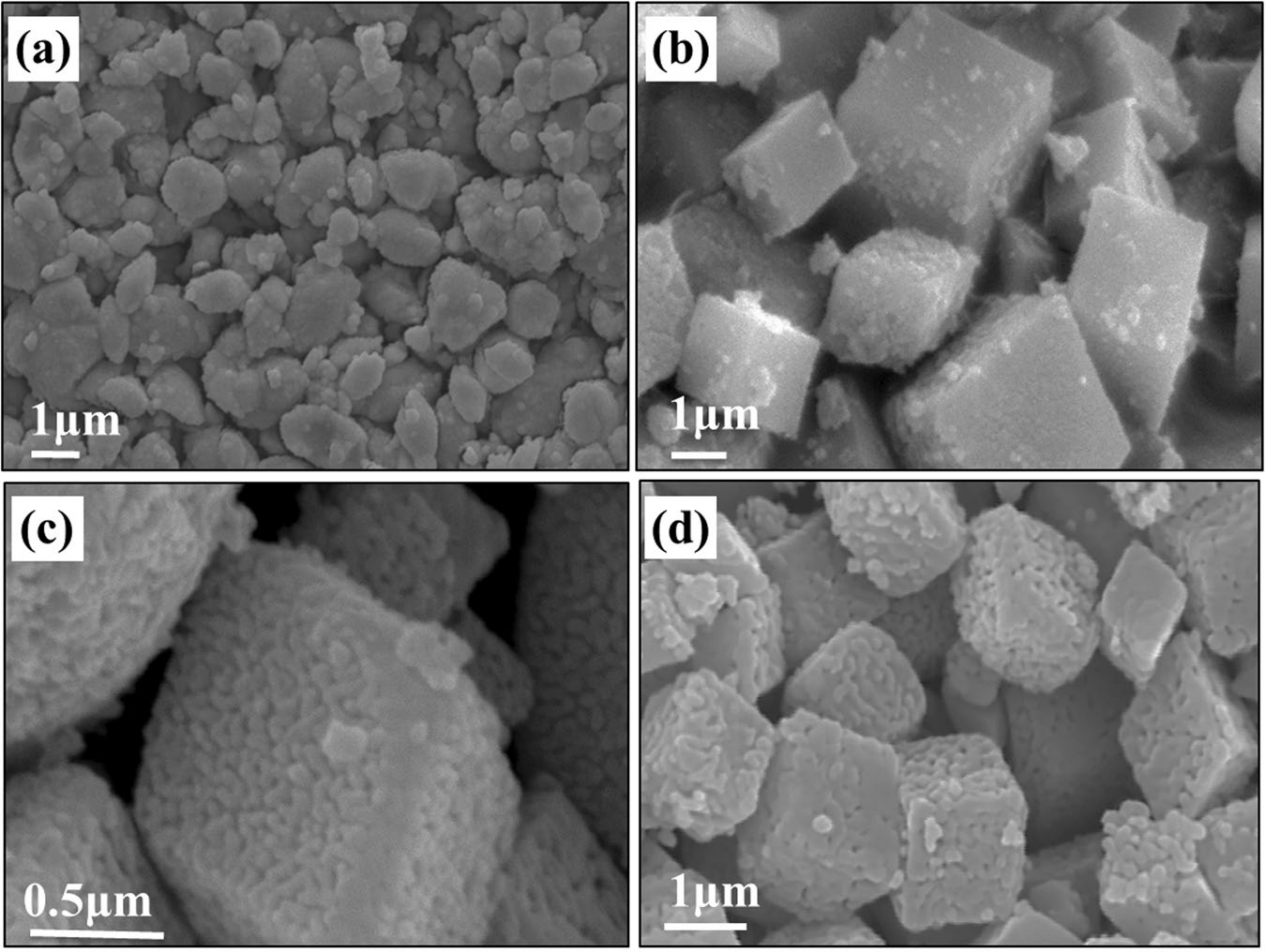
SEM images of (**a**) Mn_2_O_3_ (C600), (**b**) Mn_2_O_3_ (S500), (**c**) Mn_2_O_3_ (S600) and (**d**) Mn_2_O_3_ (S700).

Surface morphologies of MnCO_3_ (C) and MnCO_3_ (S) precursors were examined using SEM analysis, as depicted in Fig. S2. SEM analysis reveals that particles are agglomerated clusters and non-uniformly distributed with a size of 0.5–1.5 µm. It is clearly observed that Mn_2_O_3_ (C600) obtained from MnCO_3_ (C) precursor possesses irregular shape with an approximate particle size between 0.5 and 1.3 µm (Fig. 4(a)). Conversely, well-defined cubic particles of Mn_2_O_3_ were formed through the calcination of MnCO_3_ (S) precursor (Fig. 4(b–d)). Mn_2_O_3_ (S500) consists of inhomogeneous cubic particles in the range of 1.0−1.2 µm in sizes (Fig. 4(b)). Mn_2_O_3_ (S600) exhibits larger cubic particles (0.9−1.7 µm sizes) (Fig. 4(c)) with numerous nanoparticles (sub-units) observed on the surface. Moreover, cubic Mn_2_O_3_ are organised by nanosized sub-units (40−50 nm) with distinct voids between the sub-units (Fig. S3). The *d*-spacing of 0.38 nm of the sample corresponded well with (211) lattice plane of Mn_2_O_3_ (Fig. S4). Clearly, well-defined cubic particles of Mn_2_O_3_ were observed with an average size of approximately 1.1−1.7 µm, when the precursor was calcined at a high temperature of 700 °C (Fig. 4(d)). Moreover, a porous-like structure is clearly visible at the surface of the cubic at 700 °C. This type of structure is often formed if metal carbonate is used as a precursor because it releases O_2_ and CO_2_ from the interior of the metal carbonate, which possibly leads to a finer or porous structure^13,14,16^. The obtained electron microscopy results demonstrate that one-step decomposition of the synthesised MnCO_3_ precursor produces a clear facet with well-defined cubic Mn_2_O_3_ structures compared to the two-step decomposition of the commercial MnCO_3_. The hysteresis loops (Fig. S5) reveal that these materials exhibit type IV isotherm, indicating a disordered mesoporous structure with average pore diameter between 5 and 60 nm. The calculated BET specific surface area for the Mn_2_O_3_ powders are tabulated in Table S2. It is well known that nanostructures play a crucial role in electrochemical processes due to their capability to enhance mass diffusion and transportation such as electrolyte penetration or ion transport^31^. Moreover, porous-like nanostructures can allow an electrolyte to diffuse smoothly within the lattice fringes of the crystals, providing more active sites and shortened ion route. Such structures are also beneficial because they relieve the stress and buffer the volume changes caused by pulverisation and aggregation process during redox reaction^32,33^. Therefore, the structure of Mn_2_O_3_ obtained from MnCO_3_ (S) precursor is likely to enhance Na-ion storage performance.

**Figure 5:**
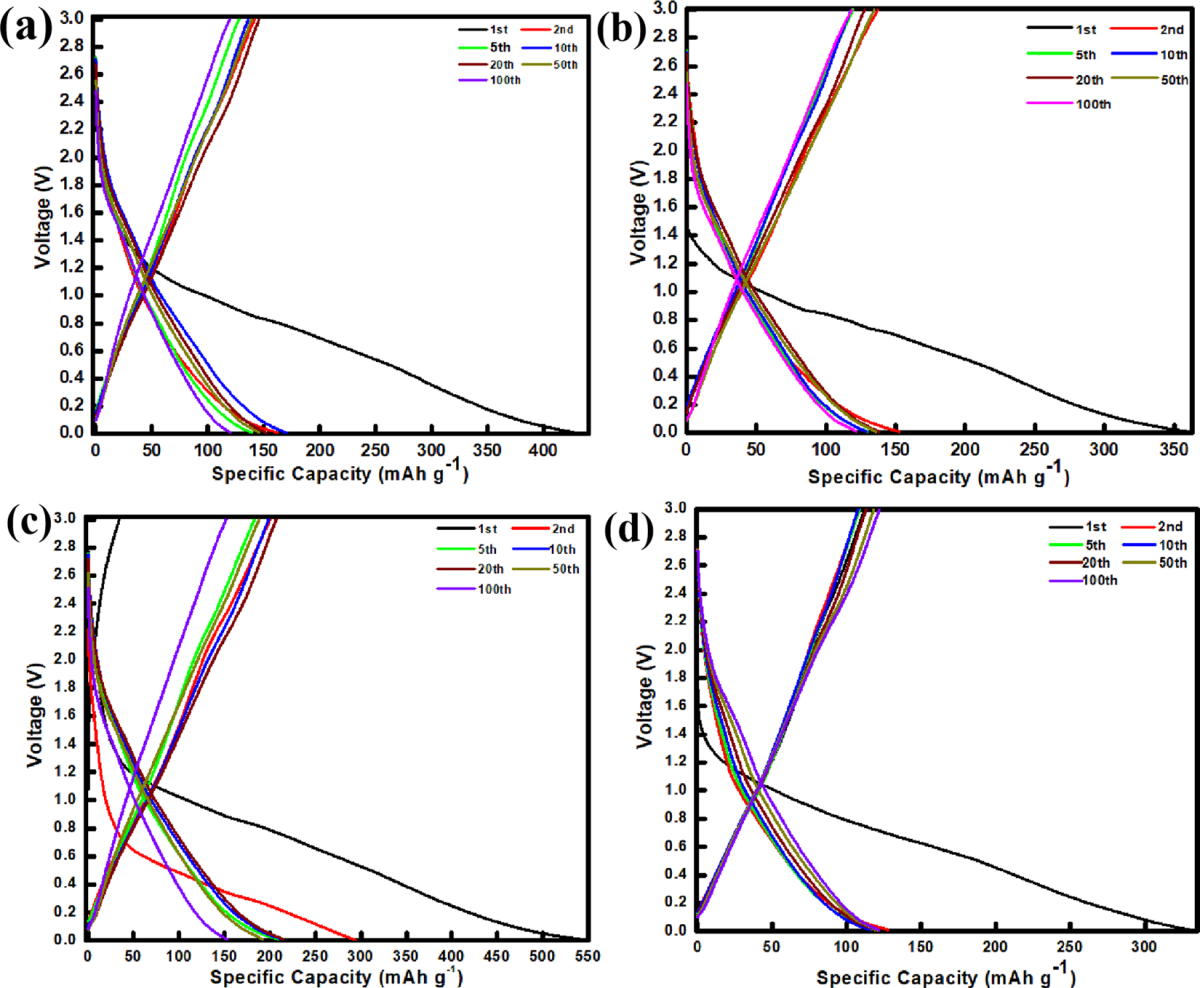
Galvanostatic charge/discharge profiles of (**a**) Mn_2_O_3_ (C600), (**b**) Mn_2_O_3_ (S500), (**c**) Mn_2_O_3_ (S600), and (**d**) Mn_2_O_3_ (S700).

The galvanostatic charge/discharge measurements of the Mn_2_O_3_ electrodes at a current density of 100 mA g^−1^ within the potential range of 0.01−3.00 V (vs. Na/Na^+^) are shown in Fig. 5. At the first cycle, irreversible capacities observed in all electrodes may be attributed to the undesirable growth of a surface passivation layer of solid electrolyte interphase (SEI). The discharge/charge potential profile of the Mn_2_O_3_ (S600) electrode was further supported by CV analysis as demonstrated in Fig. S6. However, the SEI formation plateau for the Na/Na^+^ system is not as sharp/long as compared to the Li-ion cell^34,35^. Conversely, no distinct plateau observed in the charge/discharge curves after the first cycle, typically seen and consistent with other metal oxides anode in the Na/Na^+^ system^10,12,36–38^. During cathodic process, Mn and Na_2_O were observed from the *ex-situ* XRD patterns (Fig. S7), whereas, re-formation of Mn_2_O_3_ was observed during anodic process. It is important to note that the evidence of Mn phase formation is hardly found in the XRD patterns because of significant overlap of this Mn peak with very strong peak from copper (Cu) current collector. Therefore, the formation of Mn and Na_2_O and the re-formation of Mn_2_O_3_ can be expressed by the following electrochemical reversible conversion reaction in Eq. (4).

4 $${{\rm{Mn}}}_{2}{{\rm{O}}}_{3}+6{{\rm{Na}}}^{+}+6{{\rm{e}}}^{-}\leftrightarrow 2{\rm{Mn}}+3{{\rm{Na}}}_{2}{\rm{O}}$$

**Figure 6:**
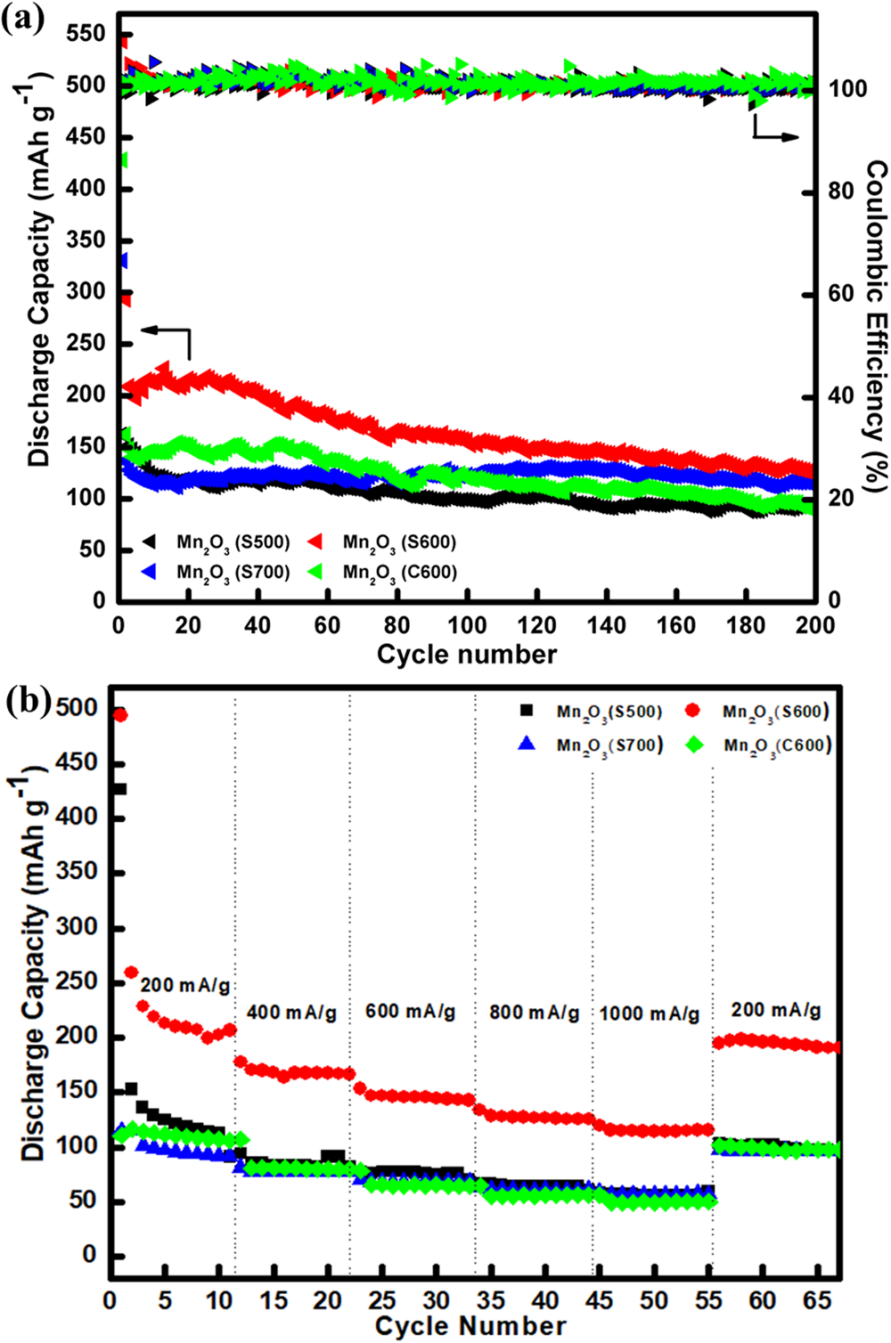
(**a**) Cycling performances and the Coulombic efficiencies up to 200 cycles at 100 mA g^−1^ and (**b**) rate capability for the Mn_2_O_3_ electrodes.

Figure 6 shows the cycling performance of the Mn_2_O_3_ electrodes. Figure 6(a) compares cycling stability and Coulombic efficiency among the electrodes measured at a current density of 100 mA g^−1^ up to 200 cycles. All electrodes exhibit high initial discharge capacity of 544 mAh g^−1^ for Mn_2_O_3_ (S600), 429 mAh g^−1^ for Mn_2_O_3_ (C600), 331 mAh g^−1^ for Mn_2_O_3_ (S700) and 163 mAh g^−1^ for Mn_2_O_3_ (S500). High initial discharge capacity could be related to the formation of SEI layer and the electrolyte decomposition itself ^39^. For Mn_2_O_3_ (S600) electrode, the discharge capacity increased after the 2^nd^ cycle and then started to decrease after ~15 cycles. Similar trends were observed for Mn_2_O_3_ (S700) electrode, where the discharge capacity increased gradually and then started to decrease after 140 cycles, which was possibly due to the activation and stabilisation processes within the electrode^40–44^. Nevertheless, the capacity depletion behaviour was noticed in Mn_2_O_3_ (S500) and Mn_2_O_3_ (C600) electrodes. At 2^nd^ cycle, the Mn_2_O_3_ (S600) electrode exhibited the highest discharge capacity of 294 mAh g^−1^ and gradually decreased to 130 mAh g^−1^ after 200 cycles. For the Mn_2_O_3_ (S700) electrode, the discharge capacity was 137 mAh g^−1^ at 2^nd^ cycle and reached 116 mAh g^−1^ after 200 cycles. In the case of Mn_2_O_3_ (S500) and Mn_2_O_3_ (C600) electrodes, the discharge capacity at the 2^nd^ cycle was 243 and 162 mAh g^−1^, respectively. After 200 cycles, both electrodes had the lowest discharge capacity, i.e. 66 mAh g^−1^ and 89 mAh g^−1^. After the initial cycle, all electrodes showed very high Coulombic efficiency of approximately 100% throughout the cycles.

Rate capability of the Mn_2_O_3_ electrodes were also measured at different charge/discharge current densities and sustained for 11 cycles for each current density (Fig. 6(b)). At the initial 11^th^ cycle, the Mn_2_O_3_ (S600) electrode delivered a high discharge capacity of 207 mAh g^−1^ at 200 mA g^−1^. When the current density increased, the electrode exhibited high retention of 166 mAh g^−1^ at 400 mA g^−1^, 143 mAh g^−1^ at 600 mA g^−1^, 126 mAh g^−1^ at 800 mA g^−1^ and 115 mAh g^−1^ at 1000 mA g^−1^. Moreover, consecutive cycling performances of Mn_2_O_3_ (S500), Mn_2_O_3_ (S700) and Mn_2_O_3_ (C600) electrodes were not as good as Mn_2_O_3_ (S600). Returning to 200 mA g^−1^ after it had been exposed to different discharge rates, the Mn_2_O_3_ (S600) electrode was able to restore the discharge capacity of 197 mAh g^−1^, which represents above 90% capacity recovery.

All the above results show that cubic-like Mn_2_O_3_ demonstrates a possible insertion/deinsertion of Na-ion with a reasonable capacity and cycling stability. The key factor that contributes to the improved performances may be offered by the special morphology of Mn_2_O_3_ itself. It is well known that the surface area is proportional to the insertion sites for ions movements^45^ and this could be ameliorated by downsizing the materials or porous architectures. The Mn_2_O_3_ synthesised here is cubic-like particles with nanoparticles (sub-units) embedded on their surfaces which in turn improve the electrochemical performances of the battery. Such Mn_2_O_3_ structure can be obtained by thermal decomposition of high quality starting metal source. Thermal decomposition of commercial MnCO_3_ produced irregular shapes of Mn_2_O_3_ whereas, hydrothermally synthesised MnCO_3_ resulted in cubic-like Mn_2_O_3_ particles. It is important to highlight that the use of glucose as reducing agent gives advantages to the precursor for growth in a required direction, thus developing well-crystallised MnCO_3_ particles via the simple route with good reproducibility. Without using any scarifying template to form porous-like structure, this method is practical for scaling-up production in the industry. Clearly, the electrochemical characteristics of Mn_2_O_3_ in Na-ion storage is promising and needs to be further explored. The cubic-like Mn_2_O_3_ with nanoparticles on its surface provides more accessible sites for electrolyte penetration into inner Mn_2_O_3_ and exposes a large area for Na-ions transportation. Meanwhile, a short ion diffusion path could facilitate the charge-transfer and greatly improve the rate capability of the Na-ions. Additionally, porous-like structure of Mn_2_O_3_ could suppress the stress created by volume changes during insertion/deinsertion process. Overall, the electrochemical activity, i.e. synthesis process, discharge capacity and rate capability, demonstrated by Mn_2_O_3_ in this study is quite impressive. The performance of Mn_2_O_3_ anode can be further improved by controlled synthesis of highly porous nanostructured with high surface area. Such a porous nanostructured needs to be integrated with conductive matrix such as surface carbon coating or hybrid formation with graphite or graphene or carbon nanotubes^46–48^. These carbon materials will not only enhance electrical conductivities of the Mn_2_O_3_ electrodes, but also will prevent agglomeration of nanostructured Mn_2_O_3_ active materials during repeated cycling, leading to much improved electrochemical performance in terms of capacity, stability, and rate capability. The findings obtained from this research create opportunities for other researchers to explore this material as an anode for Na-ion batteries.

## Materials and Methods

### Synthesis of MnCO_3_ and Mn_2_O_3_

**Scheme 1:**
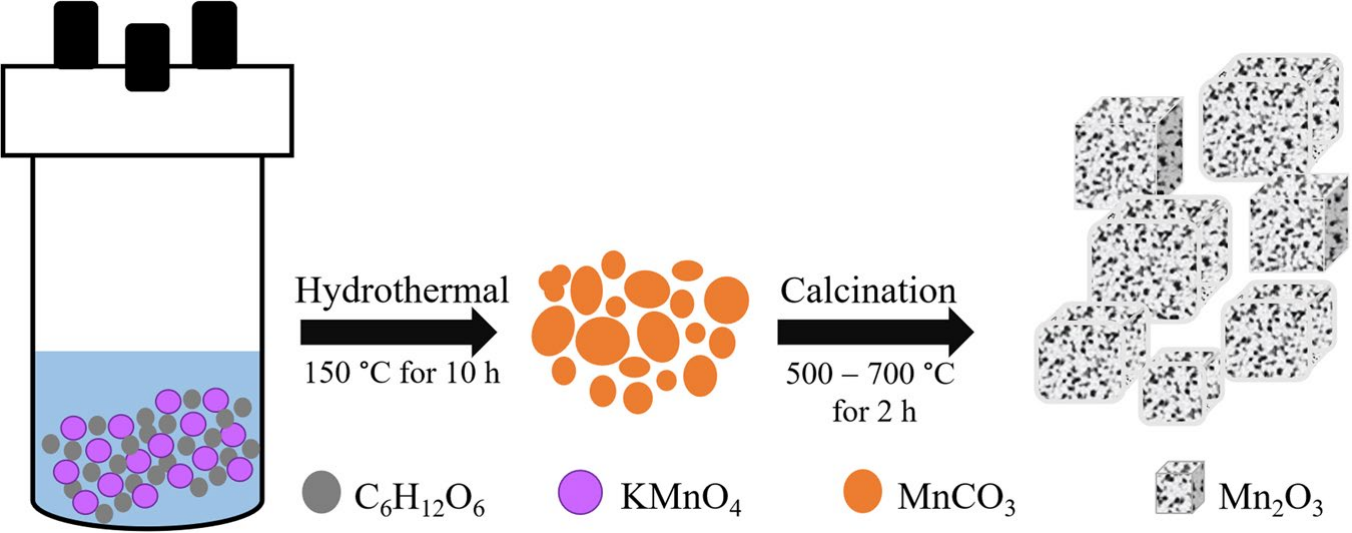
A schematic presentation for the formation of Mn_2_O_3_.

Scheme 1 shows the synthesis strategy of Mn_2_O_3_ from the starting materials. To prepare MnCO_3_, 0.3 mmol *D*-(+)-glucose (C_6_H_12_O_6_, Merck Millipore) and 0.3 mmol KMnO_4_ (Sigma-Aldrich, 97%) were dissolved in 60 ml deionised water at room temperature and stirred to form a homogeneous solution. Then, the homogeneous mixture was transferred into a 125 ml stainless steel autoclave, sealed and heated at 150 °C for 10 h. During the hydrothermal reaction, MnO_4_^−^ was reduced by glucose and Mn^2+^ ions generated, leading to the formation of MnCO_3_ according to the Eq. (5) below^49,50^.

5 $$24{{\rm{KMnO}}}_{4}+5{{\rm{C}}}_{6}{{\rm{H}}}_{12}{{\rm{O}}}_{6}=24{{\rm{MnCO}}}_{3}+6{{\rm{K}}}_{2}{{\rm{CO}}}_{3}+12{\rm{KOH}}+24{{\rm{H}}}_{2}{\rm{O}}$$

The precipitates were collected, washed several times with absolute ethanol and deionised water and dried overnight under vacuum. The dried sample is marked as (MnCO_3_ (S)). To obtain Mn_2_O_3_, MnCO_3_ (S) was calcined at 500, 600 and 700 °C in air for 2 h and denoted as Mn_2_O_3_ (S500), Mn_2_O_3_ (S600) and Mn_2_O_3_ (S700), respectively. For comparison purpose, as-received MnCO_3_ (Sigma-Aldrich, 98%) marked as MnCO_3_ (C) was also used in this study. MnCO_3_ (C) was calcined at 600 °C in air for 2 h and later denoted as Mn_2_O_3_ (C600).

### Materials characterization

The phase purity and structure of MnCO_3_ and Mn_2_O_3_ samples were determined by X-ray diffraction (XRD, Rigaku Miniflex II) with monochromatic CuKα radiation at a wavelength (λ) of 1.5406 Å. The morphology of the samples was observed through scanning electron microscopy (SEM, JOEL JSM-6360L) and transmission electron microscopy (TEM, TECNAI G2 F20) with an accelerating voltage of 200 kV. The thermogravimetric analysis (TGA) was conducted on Mettler-Toledo thermogravimetric analysis/differential scanning calorimetry (TGA/DSC 1) Star^e^ System at a heating rate of 10 °C min^−1^ in air. The Fourier transform infra-red (FTIR) spectroscopy was recorded on an IR Tracer-100. Raman spectra were collected on Raman spectroscopy (Renishaw, 532 nm radiation) extended with 0.1 power laser measurement.

### Electrochemical measurements

To investigate the electrochemical performances of Mn_2_O_3_ samples, the active materials, carbon black (Sigma-Aldrich, >99.95%) and poly(vinylidene fluoride) (PVDF, Sigma-Aldrich), in a weight ratio of 75:20:5 were dissolved in an *N*-methylpyrrolidone (NMP). The slurry was pasted onto a copper (Cu) foil with an approximate active material loading of ~ 2 mg cm^−2^. The electrodes were then dried at 100 °C overnight under vacuum. Subsequently, the electrode was cut to 1 cm × 1 cm size. Coin-type cell (CR 2032) was assembled in an Argon-filled glove box (Mbraun, Unilab, Germany) using sodium metal (Sigma-Aldrich, 99.9% trace metals basis) as the counter electrode. A Whatman glass fibre (GF/D) was used as a separator, and the electrolyte 1 M NaClO_4_ (Sigma-Aldrich, 98%), was dissolved in propylene carbonate (PC) (Sigma-Aldrich, anhydrous, 99.7%) with the addition of 5 wt.% of fluoroethylene carbonate (FEC) (Sigma-Aldrich, 99%). The cycling performance of the electrodes was conducted by Neware battery tester at room temperature.

## Conclusion

The cubic-like Mn_2_O_3_ was successfully obtained through thermal decomposition of the hydrothermally synthesised MnCO_3_. For comparison, Mn_2_O_3_ obtained through thermal conversion of commercial MnCO_3_ was also investigated. The synthesis method employed in this study offers a simple and practical approach to industrial production. A microstructure of cubic-like Mn_2_O_3_ with nanoparticles (sub-units) embedded on its surface was obtained. The electrochemical results indicate that the Mn_2_O_3_ electrode can deliver a promising discharge capacity, cyclability and rate capability during the insertion/deinsertion of Na-ions. The Mn_2_O_3_ electrode exhibited high initial discharge capacity of 544 mAh g^−1^ at 100 mA g^−1^ and reached 130 mAh g^−1^ after 200 cycles. The obtained Mn_2_O_3_ structure promotes electrolyte penetration into the interior of Mn_2_O_3_, provides large sites to facilitate fast ion transportation and thus, expedites the charge-transfer within the electrode. Therefore, the results demonstrate strong evidence for its application in Na-ion batteries.

## Supplementary information

Supplementary information.
