# Supplementary material for: Investigation on the Electrochemical Performances of Mn2O3 as a Potential Anode for Na-Ion Batteries
Source: Sci Rep. 2020 Jun 8;10:9207. doi: 10.1038/s41598-020-66148-w (PMC7280266; doi:10.1038/s41598-020-66148-w)
Supplement: Supplementary file 1 — Supplementary information. [file 41598_2020_66148_MOESM1_ESM.docx]

**Investigation on the Electrochemical Performances of Mn_2_O_3_ as a Potential Anode** **for Na-Ion Batteries**

Nor Fazila Mahamad Yusoff^a^, Nurul Hayati Idris^a,^* , Mohd Faiz Md Din^b^, Siti Rohana Majid^c^, Noor Aniza Harun^d^ and Md Mokhlesur Rahman^e,^*

*^a^ Energy Storage Research Group, Faculty of Ocean Engineering Technology and Informatics, Universiti Malaysia Terengganu 21300 Kuala Nerus, Terengganu, Malaysia*

*^b^ Department of Electrical and Electronic Engineering, Faculty of Engineering, National Defence University of Malaysia , Kem Sungai Besi, 57000 Kuala Lumpur, Malaysia*

*^c^ Center for Ionics University of Malaya, Department of Physics, Faculty of Science, University of Malaya 50603 Kuala Lumpur, Malaysia*

*^d^ Advance Nano Materials (ANOMA) Research Group, Faculty of Science and Marine Environment, Universiti Malaysia Terengganu 21300 Kuala Nerus, Terengganu, Malaysia*

*^e^ Institute for Frontier Materials, Deakin University, Waurn Ponds, Victoria 3216, Australia*

*Corresponding authors

Tel.: +60 96683185; fax: +6096683391.

E-mail address: [nurulhayati@umt.edu.my](mailto:nurulhayati@umt.edu.my) (N. H. Idris)

[m.rahman@deakin.edu.au](mailto:m.rahman@deakin.edu.au) (M. M. Rahman)

**Supporting information**

Table S1. Rietveld refinement results of the MnCO_3_ and Mn_2_O_3_. The standard errors derived from the refinements are also listed in lattice columns.

| **Sample** | ***a* (Å)** | ***c* (Å)** | **Bragg R_factor_ (%)** | **R_f factor_**  **(%)** | ***χ^2^*** |
| --- | --- | --- | --- | --- | --- |
| MnCO_3_ (C) | 4.8045 (3) | 15.6891(8) | 9.45 | 5.65 | 0.846 |
| MnCO_3_ (S) | 4.8019 (2) | 15.6798(1) | 6.70 | 4.81 | 0.736 |
| Mn_2_O_3_(C600) | 9.3996 (0) | - | 3.93 | 3.91 | 2.760 |
| Mn_2_O_3_ (S500) | 9.3990 (0) | - | 6.39 | 6.33 | 7.740 |
| Mn_2_O_3_ (S600) | 9.4043 (3) | - | 7.34 | 5.88 | 0.892 |
| Mn_2_O_3_ (S700) | 9.4057 (3) | - | 7.63 | 7.86 | 0.600 |

Table S2. The specific surface area of Mn_2_O_3_ samples.

| **Sample** | **BET surface area (m^2^/g)** |
| --- | --- |
| Mn_2_O_3_ (C600) | 13.3268 |
| Mn_2_O_3_ (S500) | 27.5512 |
| Mn_2_O_3_ (S600) | 20.2528 |
| Mn_2_O_3_ (S700) | 10.1416 |

Fig. S1.


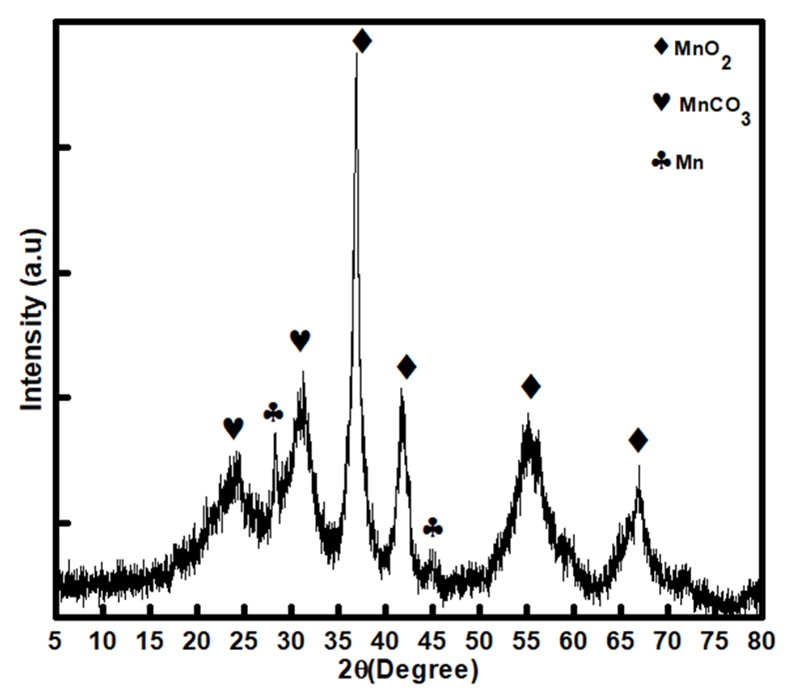


Fig. S1. XRD pattern of the MnCO_3_ (C) sample calcined at 300°C.

Fig. S2.


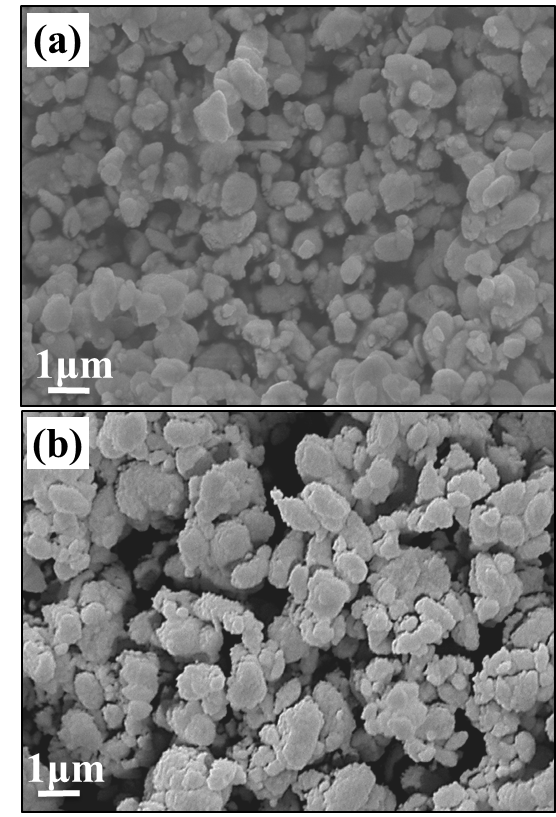


Fig. S2. SEM images of (a) MnCO_3_ (C) and (b) MnCO_3_(S).

Fig. S3.


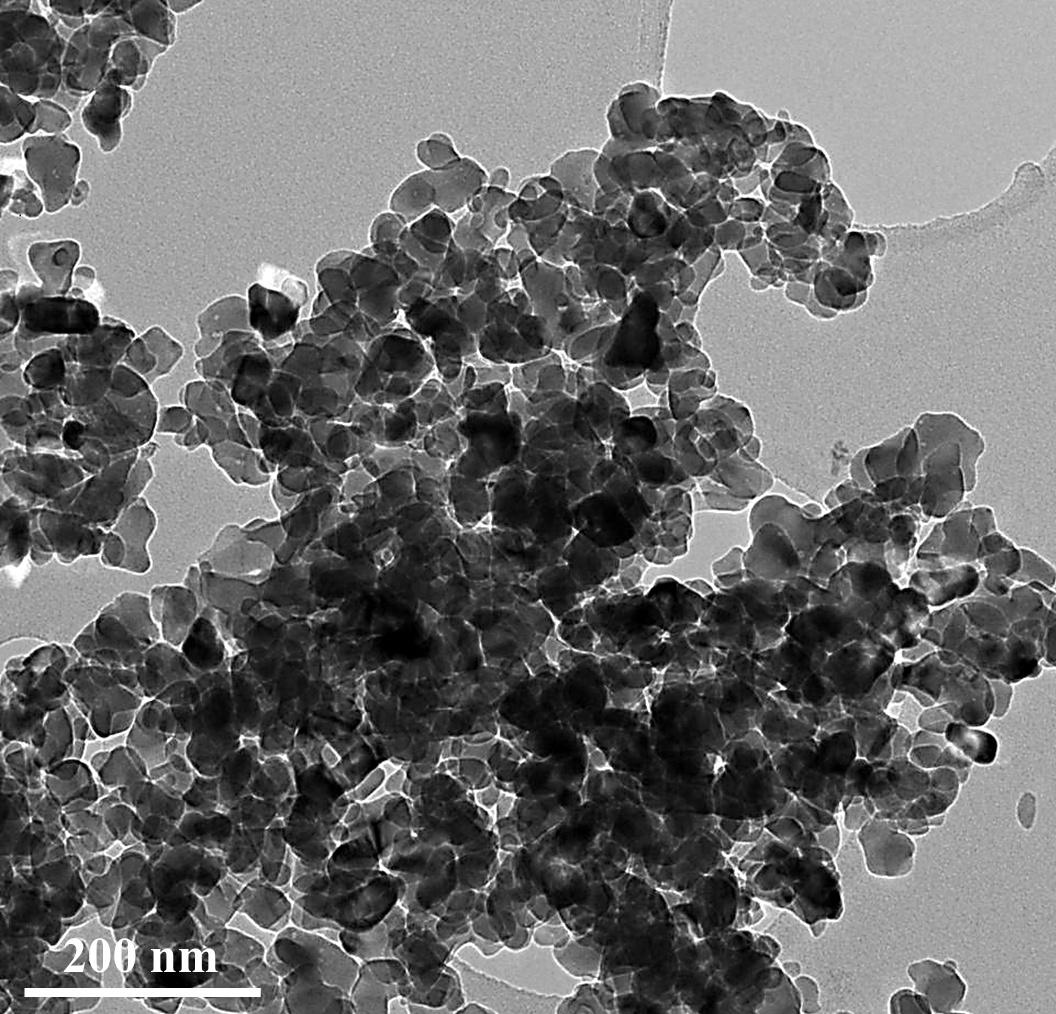


Fig. S3. TEM image of Mn_2_O_3_ (S600).

Fig. S4.


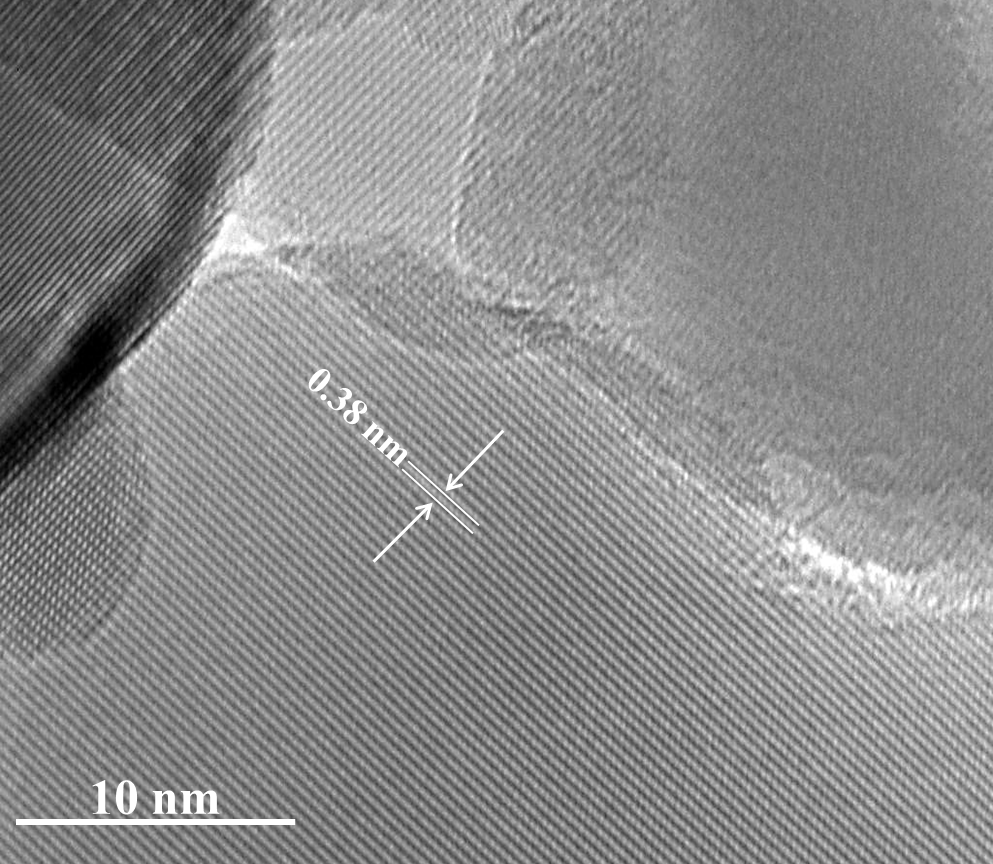


Fig. S4. HRTEM image for sample of Mn_2_O_3_ (S600).Fig. S5.


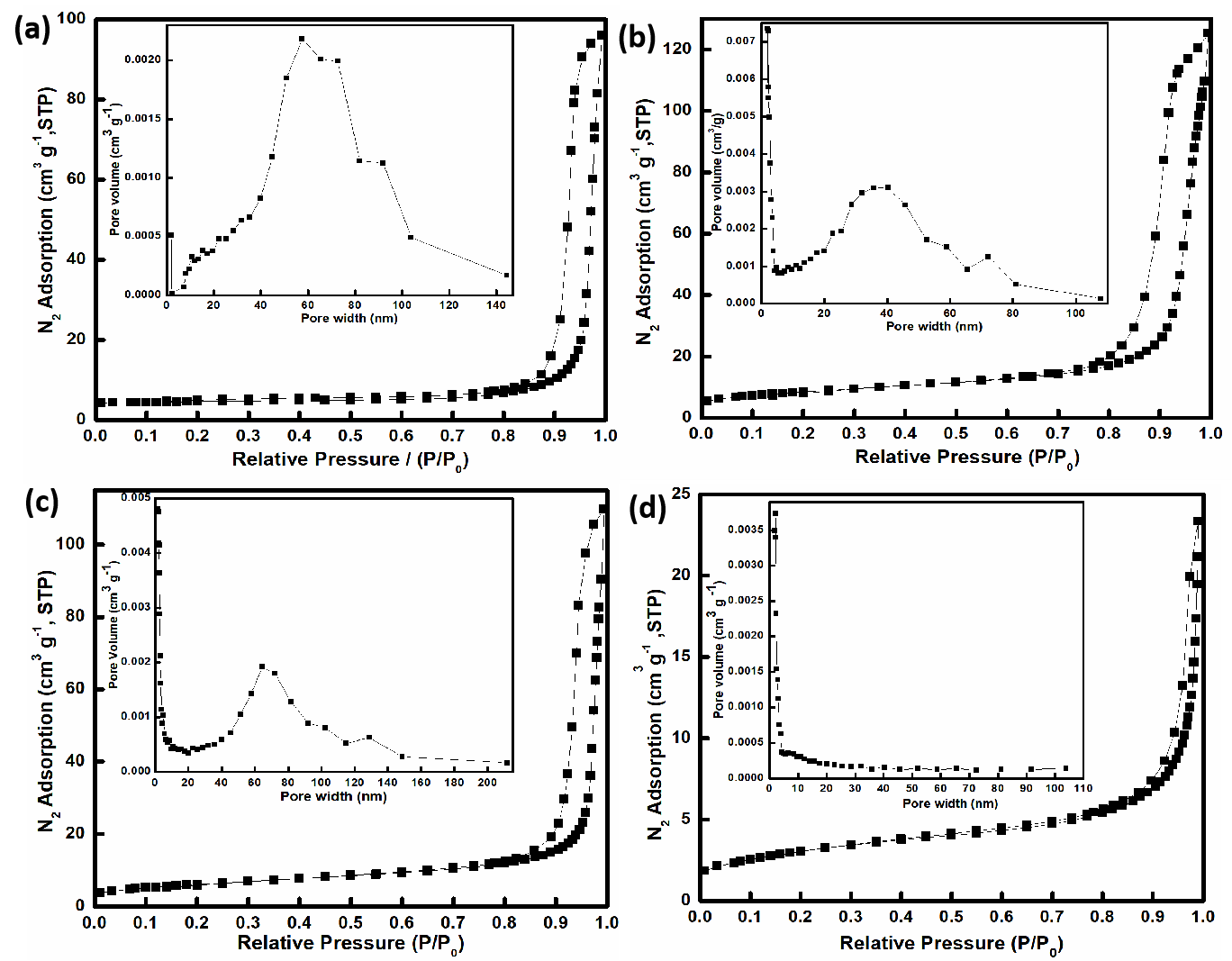


Fig. S5. N_2_ adsorption-desorption isotherms and BJH pore-size distribution curves (inset) of (a) Mn_2_O_3_ (C600), (b) Mn_2_O_3_ (S500), (c) Mn_2_O_3_ (S600), and Mn_2_O_3_ (S700).

Fig. S6.


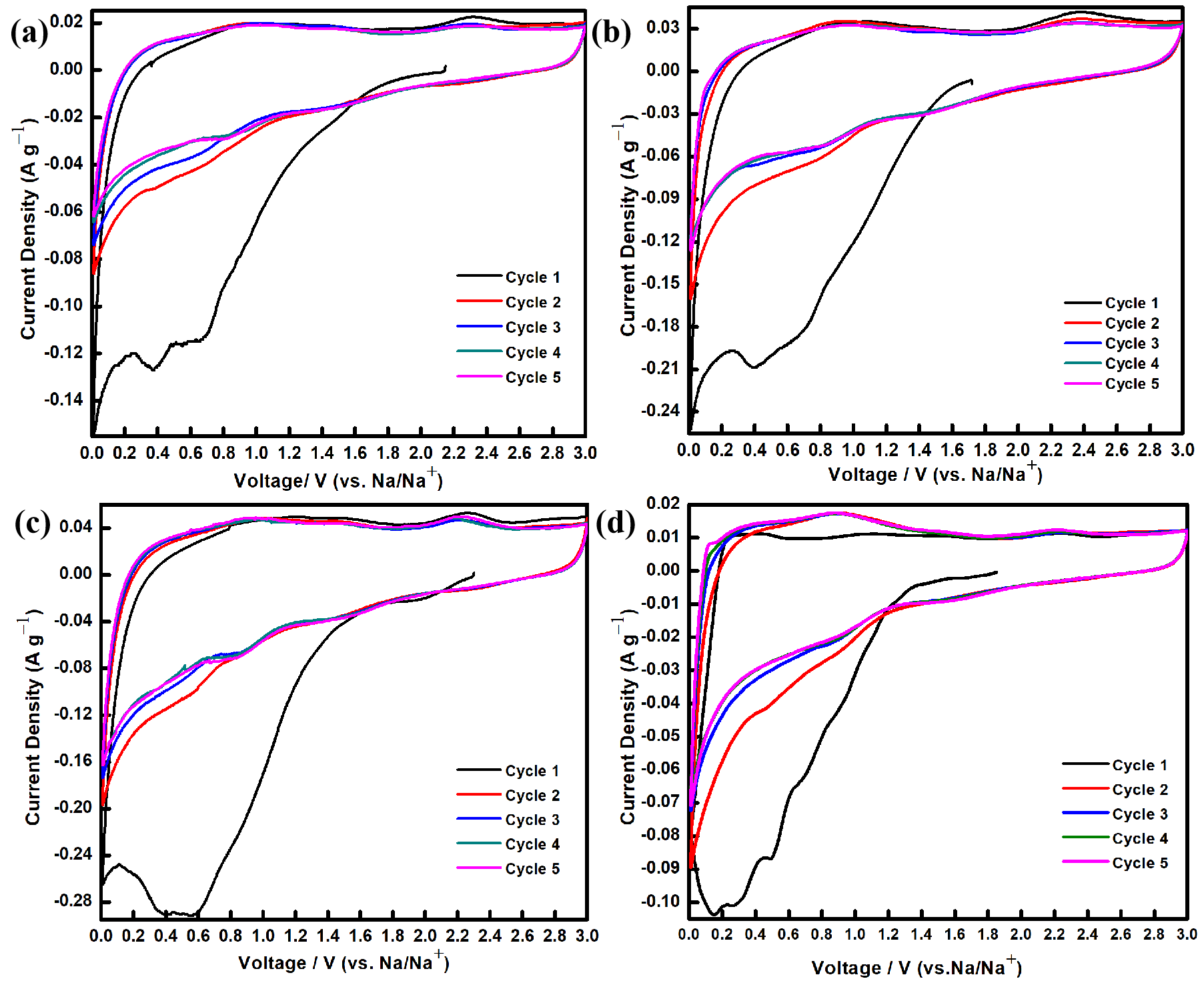


Fig. S6. CV curves of (a) Mn_2_O_3_ (C600), (b) Mn_2_O_3_ (S500), (c) Mn_2_O_3_ (S600), and (d) Mn_2_O_3_ (S700) at a scan rate of 0.3 mV s^-1^ (versus Na/Na^+^).

Fig. S7.


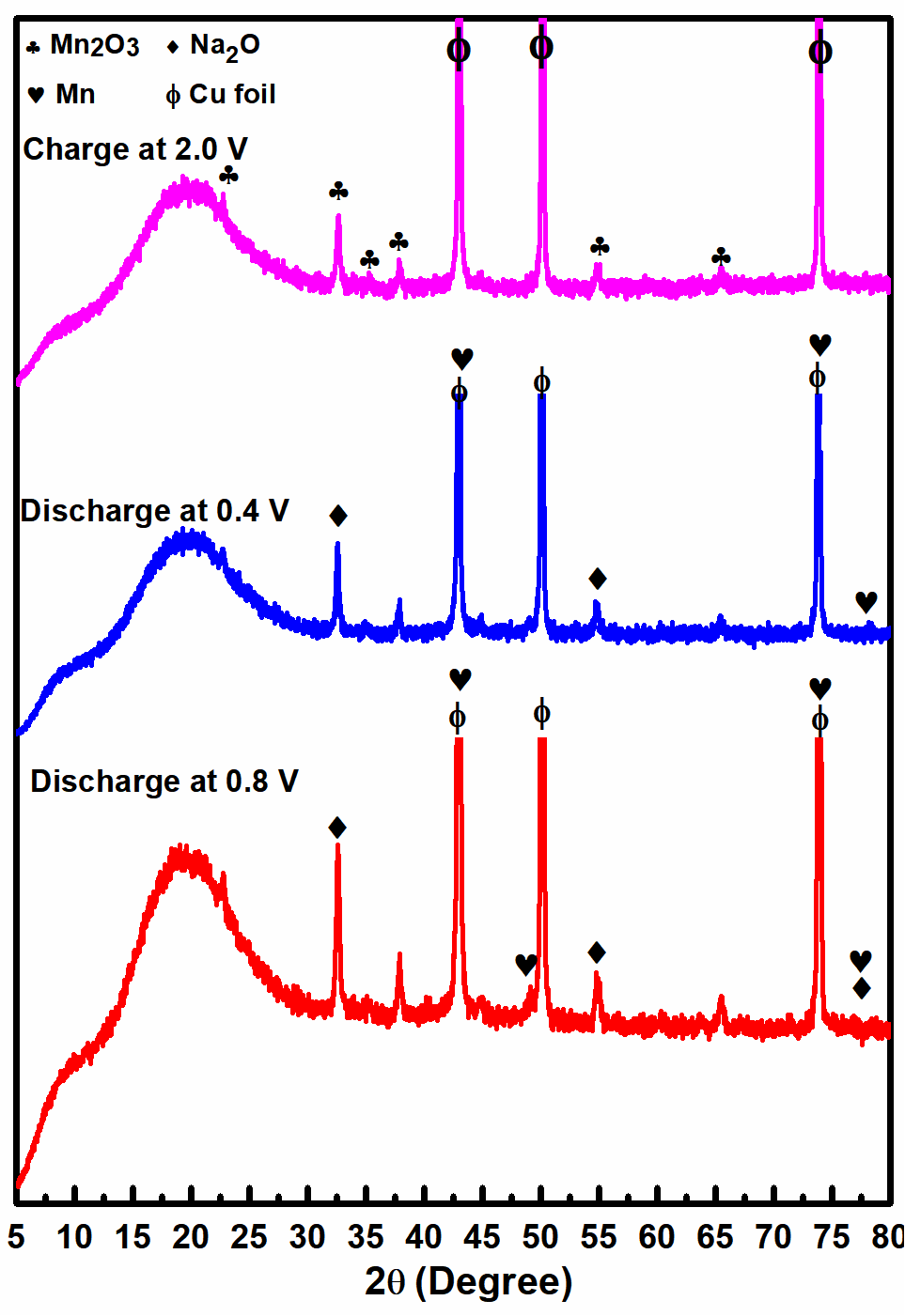


Fig. S7. Ex-situ XRD patterns of Mn_2_O_3_ electrode obtained at different charge-discharge state.
